# Inflammatory Bowel Disease Outcomes in Northern Iran: A Retrospective Cohort Study of Remission, Complications, and Treatment Strategies

**DOI:** 10.1155/bmri/5363565

**Published:** 2026-01-08

**Authors:** Poria Hoseinialiabadi, Iradj Maleki, Hafez Fakheri, Mahboobe Ebrahimi, Elham Yousefi Abdolmaleki, Tarang Taghvaei, Hajar Shokri-Afra

**Affiliations:** ^1^ Gut and Liver Research Center, Non-Communicable Disease Research Institute, Mazandaran University of Medical Sciences, Sari, Iran, mazums.ac.ir

**Keywords:** Crohn disease, inflammatory bowel disease, recurrence, remission, ulcerative colitis

## Abstract

**Background:**

Inflammatory bowel disease (IBD), encompassing Crohn′s disease (CD) and ulcerative colitis (UC), is a chronic inflammatory condition affecting the gastrointestinal tract. While significant progress has been made in managing IBD, the long‐term outcomes remain heterogeneous, prompting this cohort study.

**Methods:**

A retrospective analysis was performed on patients with IBD registered between 2000 and 2023. Clinical data, including demographics, disease activity, remission rates, therapeutic approaches, and complications, were collected.

**Results:**

Among 600 enrolled patients, 72.8% were diagnosed with UC. Both patients with UC and those with CD showed improvements in endoscopic severity by the end of follow‐up (*p* < 0.001). Clinical remission was achieved by 96.3% of patients with UC and 91.2% of those with CD during the last 6 months of follow‐up (*p* = 0.046). Disease progression occurred in 22.1% of patients with CD and 18% of those with UC (*p* = 0.100). Gastrointestinal complications were more prevalent in patients with CD (25.6% vs. 1.6%, *p* < 0.001). Relapse remained high, with 86.7% of patients with UC and 92.6% of those with CD experiencing relapses at least once during follow‐up, particularly those with severe baseline disease (*p* < 0.01). Biologic therapies were used more frequently (*p* < 0.01) and initiated earlier in patients with CD (*p* = 0.031).

**Conclusion:**

The study showed significant clinical improvement, yet many patients, particularly those with severe initial disease, had incomplete remission and frequent relapses, necessitating more effective long‐term management strategies. The distinction between CD and UC highlights the need for specialized treatments.

**Summary**

A 20‐year long‐term follow‐up was conducted despite data loss and lack of control over environmental factors due to the study′s retrospective design, as well as limitations in locally available medications. Due to our limited and incomplete data on nutritional interventions, these were not considered in this study.

## 1. Introduction

Millions of people worldwide suffer from inflammatory bowel diseases (IBDs), encompassing ulcerative colitis (UC), Crohn′s disease (CD), and IBD unclassified (IBDU), which is becoming prevalent with an expected rate of 1% within this decade. Therefore, IBD represents a significant global health concern as it significantly impacts people′s quality of life [1, 2]. The incidence pattern of IBD is rapidly rising in developing nations due to various environmental and lifestyle factors [3]. UC, a debilitating chronic disease, typically manifests with rectal bleeding, diarrhea, abdominal pain, fatigue, weight loss, and anemia [4, 5]. CD is a progressive relapsing‐remitting disease with variable anatomical location and clinical behavior. Common symptoms include abdominal pain, chronic or nocturnal diarrhea, weight loss, and fever, which serve as critical parameters for initial diagnosis [6, 7]. Although both conditions have clinical similarities, such as extraintestinal manifestations (EIMs), CD is more likely to cause complications, including bowel obstruction, perianal disease, and fistulas [8].

Clinical progression of the disease is frequently manifested through recurrent relapses, which may advance to progressive states and result in significant complications or necessitate surgical intervention. Failure to institute prompt and effective management can trigger enduring and irreversible damage [9, 10]. Inducing and maintaining remission is the primary aim of appropriate pharmacological treatment [11], although nutritional and dietary therapy had concurrently evolved as an adjunct to pharmacologic management, particularly in pediatric populations [12]. Currently available drugs include 5‐aminosalicylates (5‐ASAs), immunomodulators, biologics, and corticosteroids [13]. The severity, extent, relapse frequency, age, medications accessibility, and existence of comorbidities all affect the decision‐making process for treatment [14].

Investigating the clinical course of IBD helps in understanding the disease phases and the factors influencing its progression. This enhanced comprehension of the disease can lead to improved management and more effective treatment approaches over time. This study was aimed at investigating disease outcomes, specifically focusing on remission, complications, and treatment strategies within the target population of patients with IBD.

## 2. Materials and Methods

### 2.1. Study Design and Patients

This retrospective cohort study was conducted over a period spanning from January 1, 2000, to December 30, 2023. Data was obtained from the IBD Registry Center of Mazandaran in northern Iran, which operates as a subsidiary of the Iranian registry of Crohn′s and colitis (IRCC). The methodology for data collection in IRCC has been detailed in previous studies [15]. IBD diagnosis was based on established clinical, endoscopic, radiological, and histological criteria according to the literature [16]. A combination of IRCC data and gastroenterologist‐documented medical records was used to fulfill the study′s objectives. Patients aged 18 years or older, with complete records, no missing key variables, more than 1 year of follow‐up, and regular annual follow‐ups were included in the study. The study employed a multipoint follow‐up duration spanning three distinct time points—T1: time of diagnosis (defined as the point at which necessary diagnostic criteria for IBD diagnosis were definitively satisfied), T2: time of initiating biologic therapy (defined only for patients receiving biologics), and T3: time of ending follow‐up. Patients′ sociodemographic and basic clinical information were gathered only at T1. However, disease course and outcomes were assessed at all three time points.

### 2.2. Definitions and Disease Classification

UC extent was categorized by Montreal classification into proctitis, left‐sided colitis, and pancolitis. CD was classified by location as ileal, colonic, ileocolic, and upper gastrointestinal (GI) [17]. Disease progression is defined as an increase in the extent of bowel involvement over time. Endoscopic severity, a definition for disease activity, is assessed using the Mayo endoscopic score for UC [18] and classified as normal mucosa (inactive disease), mild activity (erythema, decreased vascular pattern, and mild friability), moderate activity (marked erythema, absent vascular pattern, friability with erosions), and severe activity (spontaneous bleeding and large ulcerations). The simple endoscopic score for Crohn′s disease (SES‐CD) defined CD activity into four levels [19] as 0–2 = remission, 3–6 = mild, 7–15 = moderate, and >15 = severe. Patients without disease symptoms and who did not require further intensive treatment were considered to be in clinical remission [20]. GI complications were defined as the occurrence of perianal abscess, perianal fistula, bowel obstruction, or megacolon. Relapse is characterized by a deterioration in symptoms, laboratory findings, or endoscopic findings following a temporary improvement, leading to the need for more intensive medical and/or surgical intervention [20–23]. Relapse rates were also evaluated at 5‐year intervals in patients who had long‐term follow‐up.

### 2.3. Ethical Consideration

The protocol of this study was approved by the Ethics Committee of Imam Khomeini Hospital, Mazandaran University of Medical Sciences (IR.MAZUMS.REC.1402.667). Ethical principles following relevant guidelines and regulations were considered by researchers at all stages of the study.

### 2.4. Statistical Analysis

Descriptive statistics were applied for demographic data. Results are reported as mean ± SD for quantitative variables and frequency (percentage) for qualitative variables. Analysis of variance (ANOVA) was performed to assess statistical differences between disease subgroups (UC, CD, and IBDU). A chi‐square test was used to compare differences of qualitative/categorical variables. Paired comparisons between T1 and T3 were performed by Wilcoxon signed ranks test or paired *t*‐test. IBM SPSS Statistics for Windows Version 27.0.1 (IBM Corp., Armonk, New York, United States) and GraphPad Prism (V.10.4.1, GraphPad Inc., United States) were used for statistical analysis and illustrations, respectively. Statistically significant values are as *p* < 0.05.

## 3. Results

### 3.1. Study Population

Of the 746 patients registered with IBD, 600 met the inclusion criteria, while 146 were excluded for not fulfilling these criteria. As shown in Table 1, most of the cases were diagnosed as UC (72.8%). Patients in the three subgroups did not differ in age (*p* = 0.094). Patients with CD were diagnosed at a younger age; however, no difference was observed between subgroups (*p* = 0.071). The mean follow‐up duration was approximately 8 years, ranging from 1 to 24 years. Most patients were female (57.2%) and nonsmokers (96.7%), with no difference between groups (*p* = 0.410, *p* = 0.309, respectively). Among the cohort, 0.5% underwent liver transplantation following hepatic cirrhosis due to advanced primary sclerosing cholangitis (PSC). In addition, 1.7% had GI cancer, 2% had cytomegalovirus (CMV) colitis, and 0.7% had *Clostridioides difficile* (*C. diff*) infection.

**Table 1:** Clinical characteristics and demographic features of patients with IBD at diagnosis (T1).

**Variables**	**Total patients** **N** (*%*) = 600 **(100)**	**Ulcerative colitis (UC)** **N** (*%*) = 437 **(72.8)**	**Crohn’s disease (CD)** **N** (*%*) = 136 **(22.7)**	**IBD unclassified (IBDU)** **N** (*%*) = 27 **(4.5)**	**Sig.** ^∗^
Age, years (mean ± SD)	45.46 ± 13.85	46.20 ± 13.94	43.28 ± 13.11	44.56 ± 15.28	0.094
Age at diagnosis, years (mean ± SD)	35.67 ± 13.55	36.11 ± 13.67	33.59 ± 12.83	38.96 ± 14.31	0.071
Duration^a^, years (mean ± SD)	8.19 ± 5.32	8.41 ± 5.46	8.23 ± 4.94	4.41 ± 3.10	**< 0.001**
Gender (male), *N* (%)	257 (42.8)	180 (41.2)	64 (47.1)	13 (48.1)	0.410
Marital status (married), *N* (%)	280 (80)	201 (81.4)	62 (79.5)	17 (68)	0.279
Smoking, *N* (%)	20 (3.3)	12 (2.7)	6 (4.4)	2 (7.4)	0.309
Residence (urban), *N* (%)	559 (93.3)	410 (94)	124 (91.2)	25 (92.6)	0.50
CMV colitis, *N* (%)	12 (2.0)	11 (2.5)	1 (0.7)	0	0.323
*C. diff* infection, *N* (%)	4 (0.7)	4 (0.9)	0	0	—
Cancer incidence, *N* (%)	20 (3.3)	11 (1.8)	9 (1.5)	0	0.401
GI cancer	10 (1.7)	5 (1.1)	5 (3.7)	0	0.103
Colorectal cancer	4 (0.7)	3 (0.7)	1 (0.7)	0	0.908
Liver transplantation, *N* (%)	3 (0.5)	2 (0.5)	1 (0.7)	0	0.860
Extension and location, *N* (%)					
Upper GI (L4)	0 (0)	N/A	0 (0)	0 (0)	**< 0.001**
Ileocolic (L3)	81 (13.6)	N/A	78 (57.4)	3 (11.5)	
Colonic (L2)	22 (3.7)	N/A	22 (16.2)	N/A	
Ileal (L1)	35 (5.9)	N/A	36 (26.5)	0 (0)	
Pancolitis (E3)	135 (22.7)	127 (29.3)	N/A	8 (30.8)	
Left‐sided colitis (E2)	195 (32.8)	182 (41.9)	N/A	13 (50.0)	
Proctitis (E1)	127 (21.3)	125 (28.8)	N/A	2 (7.7)	
First‐line treatment, *N* (%)					
5‐Aminosalicylate	577 (97.3)	431 (98.9)	120 (92.3)	26 (96.3)	**< 0.001**
Immunosuppressive	228 (38.4)	143 (32.8)	83 (62.8)	2 (7.4)	**< 0.001**
Corticosteroid	159 (26.8)	99 (22.7)	57 (43.8)	3 (11.1)	**< 0.001**
Biologic	5 (0.8)	2 (0.5)	3 (2.3)	0	0.114

Abbreviations: *C. diff*, *Clostridioides difficile*; CMV, cytomegalovirus; GI, gastrointestinal; N/A, not applicable.

^a^Follow‐up duration.

^∗^Significant values (< 0.05) are shown in bold.

### 3.2. Clinical Presentation

Table 2 shows the clinical presentations that may occur during follow‐up in more detail of initial presentations, EIMs, concurrent diseases, and nonspecific disease‐related manifestations. At T1, patients with UC exhibited a higher prevalence of rectal bleeding and bloody diarrhea (80.2%), whereas patients with CD more frequently presented with abdominal pain (58.6%) and diarrhea (53.4%) (*p* < 0.001). Hepatic involvement was the most frequent EIM in both subgroups of UC (6.6%) and CD (5.9%), followed by rheumatologic manifestations (5.7% and 4.4%, respectively) and dermatologic manifestations (0.7% and 2.9%, respectively). However, there was no significant difference in the frequency of any of the EIMs between subgroups. Other manifestations like cholelithiasis, weight loss, and fever were more common in patients with CD (*p* < 0.05).

**Table 2:** Clinical presentations and extraintestinal manifestations.

	**Total**	**Ulcerative colitis (UC)**	**Crohn’s disease (CD)**	**IBD unclassified (IBDU)**	**Sig.** ^∗^
Initial presentation, *N* (%)					
Diarrhea	212 (35.9)	128 (29.8)	71 (53.4)	13 (48.1)	**< 0.001**
Constipation	29 (4.9)	20 (4.7)	8 (6.0)	1 (3.7)	0.781
Rectal bleeding	427 (72.4)	345 (80.2)	61 (45.9)	21 (77.8)	**< 0.001**
Abdominal pain	240 (40.7)	146 (34.0)	78 (58.6)	16 (59.3)	**< 0.001**
Mucoid stool	155 (26.3)	112 (26.0)	32 (24.1)	11 (40.7)	0.195
Perianal fistula	3 (0.5)	0	3 (2.3)	0	**0.006**
Perianal fissure	14 (2.4)	6 (1.4)	8 (6.0)	0	**0.007**
Perianal abscess	3 (0.5)	0	3 (2.3)	0	**0.006**
IBD‐related extraintestinal manifestation, *N* (%)					
Hepatic	39 (6.5)	29 (6.6)	8 (5.9)	3 (11.1)	0.935
Autoimmune hepatitis	3 (0.5)	3 (0.7)	0	0	
PSC	36 (6.0)	26 (5.9)	8 (5.9)	2 (7.4)	
Rheumatologic	31 (5.2)	25 (5.7)	6 (4.4)	0	0.219
Nonspecific arthritis	17 (2.8)	13 (2.9)	4 (2.9)	0	
AS	6 (1)	5 (1.1)	1 (0.7)	0	
RA	8 (1.3)	7 (1.6)	1 (0.7)	0	
Dermatologic	7 (1.2)	3 (0.7)	4 (2.9)	0	0.064
Pyoderma‐gangrenosum	2 (0.3)	1 (0.2)	1 (0.7)	0	
Erythema nodosum	2 (0.3)	2 (0.5)	0	0	
Psoriasis	3 (0.5)	0	3 (2.2)	0	
Concurrent diseases					
Fatty liver, *N* (%)	70 (11.7)	52 (11.9)	17 (12.5)	1 (3.7)	0.412
Cholelithiasis, *N* (%)	19 (2.3)	9 (2.1)	9 (6.6)	1 (3.7)	**0.029**
Nephrolithiasis, *N* (%)	14 (2.3)	10 (2.3)	4 (2.9)	0	0.647
Nonspecific disease‐related manifestations					
Weight loss, *N* (%)	40 (6.8)	19 (4.4)	21 (15.8)	0	**< 0.001**
Pruritus, *N* (%)	6 (1.0)	5 (1.2)	1 (0.8)	0	0.794
Fever, *N* (%)	13 (2.2)	3 (0.7)	10 (7.4)	0	**< 0.001**
Malaise, *N* (%)	9 (1.5)	4 (0.9)	4 (3.0)	1 (3.7)	0.149
Arthralgia, *N* (%)	15 (2.5)	9 (2.1)	6 (4.4)	0	0.214

Abbreviations: AS, ankylosing spondylitis; PSC, primary sclerosing cholangitis; RA, rheumatoid arthritis.

^∗^Significant values (< 0.05) are shown in bold.

### 3.3. Disease Extension

At T1 (Table 1), the most common extension in patients with UC was left‐sided colitis (41.7%), while ileocolic (57.4%) was the predominant form among those with CD. An improvement toward normal condition in disease extension was found among patients with UC (Table 3) (T1 vs. T3, *p* < 0.001). The same pattern was observed for disease localization in patients with CD (results not shown in Tables 1 and 3).

**Table 3:** Disease pattern during follow‐up.

		**Total patients**	**Ulcerative colitis (UC)**	**Crohn’s disease (CD)**	**IBD unclassified (IBDU)**	**Sig.** ^∗^
IBD behavior and outcome						
Extension and location, *N* (%)						—^$^
Ileocolic (L3)	T2	27 (16.2)	N/A	23 (30.3)	0 (0)	
	T3	24 (6.8)	N/A	17 (22.4)	1 (8.3)	
Colonic (L2)	T2	14 (8.4)	N/A	14 (20.6)	N/A	
	T3	11 (3.1)	N/A	11 (14.5)	N/A	
Ileal (L1)	T2	27 (16.2)	N/A	27 (39.7)	0	
	T3	19 (5.4)	N/A	17 (22.4)	2 (16.7)	
Pancolitis (E3)	T2	43 (25.7)	43 (43.9)	N/A	0	
	T3	52 (14.7)	49 (18.4)	N/A	3 (25)	
Left‐sided colitis (E2)	T2	43 (25.7)	42 (42.9)	N/A	1 (100)	
	T3	84 (23.7)	79 (29.7)	N/A	4 (33.3)	
Proctitis (E1)	T2	13 (7.8)	13 (13.3)	N/A	0	
	T3	79 (22.3)	78 (29.3)	N/A	1 (8.3)	
Normal endoscopy	T2	0	0	0	0	
	T3	86 (24.3)	60 (22.6)	25 (32.9)	1 (8.3)	
Progression, *N* (%)		85 (19.7)	57 (18.0)	23 (22.1)	5 (41.7)	0.100
Surgery, *N* (%)		12 (2)	6 (1.4)	6 (4.4)	0	0.066
GI complications, *N* (%)		42 (7)	7 (1.6)	35 (25.7)	0	**< 0.001**
Perianal abscess		14 (33.3)	2 (28.6)	12 (34.3)	0	0.141
Perianal fistula		25 (59.5)	4 (57.1)	21 (60)	0	
Obstruction		2 (4.8)	0	2 (5.7)	0	
Megacolon		1 (2.4)	1 (14.3)	0	0	
Clinical remission		570 (95.0)	421 (96.3)	124 (91.2)	25 (92.6)	**0.046**
Relapse, *N* (%)						
First 5 years of follow‐up		296 (71.2)	213 (69.2)	77 (77)	6 (75)	0.313
First 10 years of follow‐up		189 (87.9)	144 (86.7)	44 (91.7)	1 (100)	0.611
Maximal follow‐up		522 (87.4)	377 (86.7)	125 (92.6)	20 (74.1)	**0.019**
Medications						
Biologic users, *N* (%)		180 (30)	105 (24)	74 (54.4)	1 (3.7)	**< 0.001**
Adalimumab		117 (65)	73 (69.5)	44 (59.5)	0	0.150
IFX		63 (35)	32 (30.5)	30 (40.5)	1 (100)	
Time to first biologic therapy initiation, years (mean ± SD)		5.91 ± 4.88	6.68 ± 5.05	4.88 ± 4.44	1	**0.031**
Biologic use duration, years (mean ± SD)		4.79 ± 2.65	4.51 ± 2.59	5.22 ± 2.70	2	0.125
Biologic resistance, *N* (%)		34 (29.1)	27 (42.2)	7 (13.5)	0	**0.003**
Alternative biologic therapy, *N* (%)		65 (36.1)	39 (37.1)	26 (35.1)	0	0.725
Adalimumab		34 (52.3)	14 (35.9)	20 (76.9)	0	—^$^
IFX		13 (20)	7 (17.9)	6 (23.1)	0	
Tofacitinib		18 (27.7)	18 (46.2)	N/A	N/A	

Abbreviations: GI, gastrointestinal; IBD, inflammatory bowel disease; IFX, infliximab; N/A, not applicable; T2, the time before initiating biologic therapy; T3, the time at the end of follow‐up.

^∗^Significant values (< 0.05) are shown in bold.

^**$**^Statistical analysis was not performed as some definitions were missing for subgroups, and the cells remained blank.

### 3.4. Endoscopic Severity Pattern

**Figure 1:**
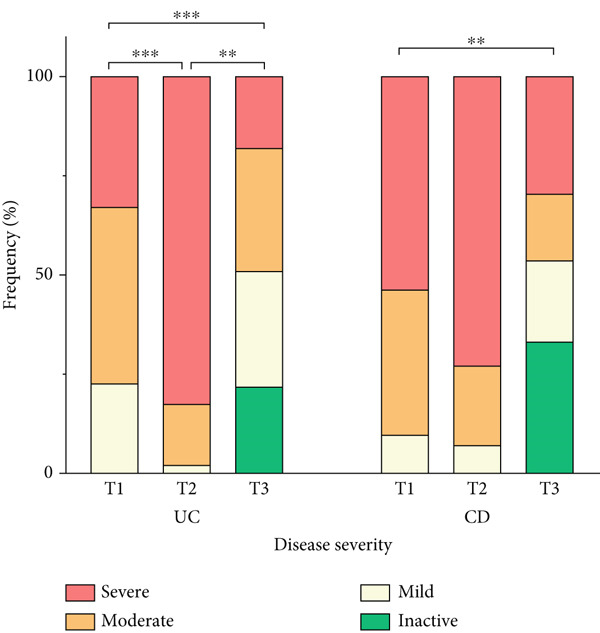
Endoscopic severity pattern during follow‐up. Columns represent the time of patients′ assessment: T1, time at diagnosis; T2, time of initiating biologic; T3, time of ending follow‐up. Stars display pairwise comparison between follow‐up times as  ^∗∗^ *p* < 0.01 and  ^∗∗∗^ *p* < 0.001 using Wilcoxon test.

As shown in Figure 1, disease severity reduced during the follow‐up period (UC: *p* < 0.001 and CD: *p* < 0.029). Endoscopic findings showed that, at T1, 44.4% and 23.9% of patients with UC exhibited moderate and severe disease, respectively. By T3, these figures decreased to 31% and 17.9%, with 21.9% presenting inactive disease. A comparable trend was observed in patients with CD, where 37% and 53.3% had moderate and severe severity at T1 decreasing to 16.7% and 29.5% at T3, respectively. Furthermore, 33.3% were classified as inactive disease at T3. Pairwise comparisons showed significant improvement in endoscopic severity between three time points in patients with UC, but in those with CD, there was a significant difference only between T1 and T3 (Figure 1).

### 3.5. Medical Treatment

Table 1 shows that 5‐ASA was the most frequently prescribed first‐line treatment in almost all patients (*p* < 0.001); however, immunomodulators and corticosteroids were used more among patients with CD (*p* < 0.001). Among immunomodulator users, azathioprine was the most common choice for both subgroups of UC (65.7%) and CD (88.2%). Methotrexate and 6‐mercaptopurine were used less frequently in patients (data not shown).

As shown in Table 3, biologic therapy (anti‐TNF agent) was initiated in 180 (30%) patients with IBD. Among them, 24% of those with UC and 54.4% of those with CD (*p* < 0.001) received biologic treatment at a mean duration of 6.68 and 4.88 years after diagnosis, respectively (*p* = 0.031). However, treatment duration was comparable between subgroups (*p* = 0.125). Adalimumab was the most commonly used anti‐TNF agent in both subgroups (69.5% and 59.5%, respectively) (*p* = 0.150). Higher rates of resistance, occurring as either primary nonresponse or secondary loss of response, were observed in anti‐TNF users with UC (42.2% vs. 13.5%, *p* = 0.003). Thus, resistance necessitated switching to alternative biologic agents in 65 cases with IBD (36.1%), with tofacitinib most frequently prescribed for cases with UC (46.2%) and adalimumab for those with CD (76.9%).

### 3.6. Disease Behavior and Clinical Outcome

Table 3 shows an improvement in the extent of bowel involvement at T3 compared to T1 (Table 1), with 86 patients (24.3% of the total) achieving normal endoscopic findings. Nonetheless, 18% and 22.1% of patients with UC and CD experienced progression to more extensive forms of the disease during follow‐up (*p* = 0.100). GI complications were more common in patients with CD (25.6% [*n* = 35]  vs. 1.6% [*n* = 7], *p* < 0.001), with anal fistula being the most frequent complication in both subgroups of CD (*n* = 21) and UC (*n* = 4). Of those with complications, a total of 12 patients underwent surgical procedures: 5 total colectomies (all in the UC subgroup), 5 partial colectomies (4 in the CD subgroup), and 2 ileal resections (in the CD subgroup). However, 96.3% of patients with UC and 91.2% of those with CD reported achieving clinical remission during the last 6 months of their follow‐up period (*p* = 0.046).

Patients with UC (86.7%) and CD (92.6%) experienced at least one relapse during the follow‐up period (*p* = 0.019). However, the relapse rate was lower in the first 5 years of disease (69.2% vs. 77%), then progressively increased, and by 10 years reached levels similar to the overall relapse rates (86.7% vs. 91.7%). Figure 2 shows that the frequency of relapses was also associated with disease severity (based on endoscopy findings at T1). Accordingly, in both subgroups of UC and CD, those with severe disease experienced more relapses during the follow‐up period (*p* < 0.001 and *p* = 0.002, respectively).

Figure 2Relapse frequency in patients with IBD. (a, c) The mean number of annual relapses during follow‐up period is displayed by initial endoscopic intensity (at T1) in patients with UC and CD. (b, d) Relapse ratio is shown at different endoscopic intensities (based on T1). The relapse ratio was determined by dividing the number of relapses by the duration of follow‐up, which represents the probability of annual relapse. The frequency of flare during IBD follow‐up and between three disease severities was compared using the analysis of variance (ANOVA), and statistical differences are shown as *p* values. Abbreviations: UC, ulcerative colitis; CD, Crohn′s disease; Duration, duration of follow‐up; Relapse ratio, relapse/duration.

**Figure:**
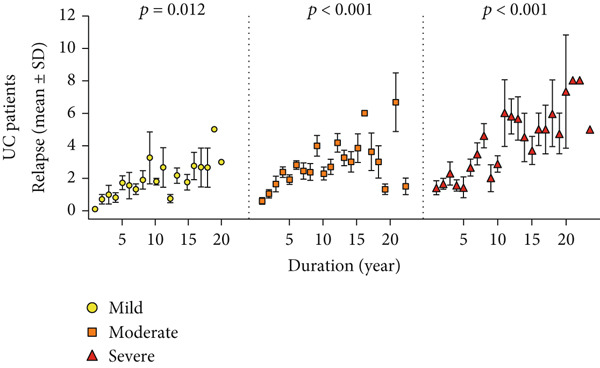
(a)

**Figure:**
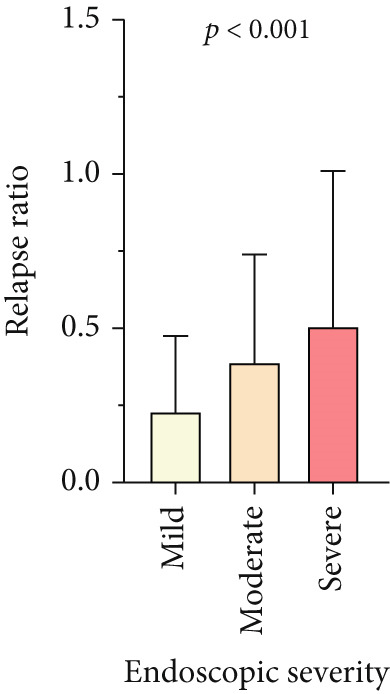
(b)

**Figure:**
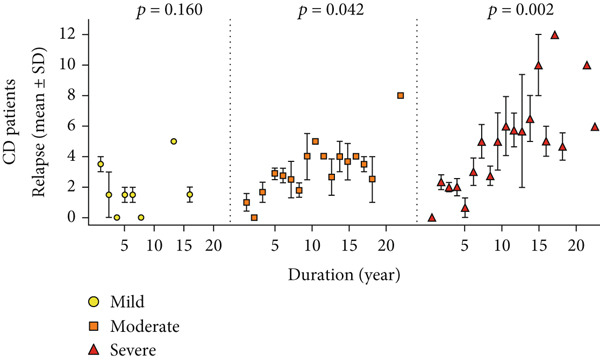
(c)

**Figure:**
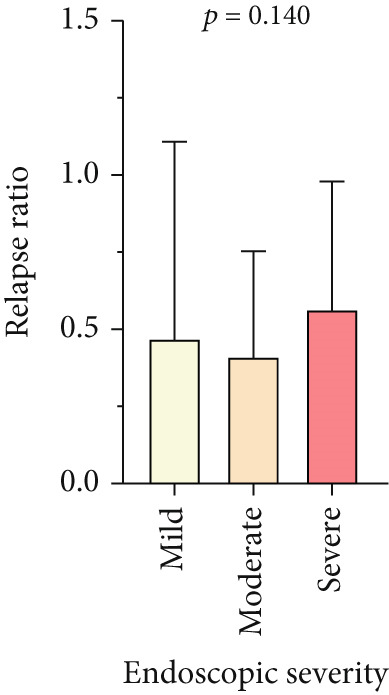
(d)

### 3.7. Laboratory Findings

As shown in Figure 3, CRP levels (milligrams/deciliter) decreased by the end of follow‐up, but this change was not significant in patients with UC, contrasting with the findings in those with CD (T1: 10.28 ± 14.92 vs. T3: 4.26 ± 4.95, *p* = 0.006). Our results also showed a decrease in the final levels of FC (microgram/gram) in patients with UC (T1: 815.37 ± 541.41 vs. T3: 222.77 ± 364.60, *p* = 0.003), whereas this reduction was not statistically significant in those with CD. Interestingly, FC was significantly elevated in both subgroups at T2, when disease exacerbation necessitated biologic initiation, possibly reflecting the severity of inflammation, but it decreased significantly compared to the final levels at T3. Hb levels showed no significant changes during treatment in both subgroups of UC and CD. However, WBC (×cells/*μ*L) exhibited a similar decrease pattern in both subgroups of UC (T1: 8177 ± 2977 vs. T3: 6643 ± 1977, *p* < 0.001) and CD (T1: 7549 ± 2072 vs. T3: 6233 ± 1832, *p* < 0.001).

Figure 3(a–d) Laboratory findings between three time points. Each column indicates patients′ assessment time: T1, time at diagnosis; T2, time of initiating biologic; T3, time of ending follow‐up. Pairwise comparison between follow‐up times was performed using the Wilcoxon test, and stars show the statistical differences as  ^∗∗^ *p* < 0.01. Abbreviations: UC, ulcerative colitis; CD, Crohn′s disease; CRP, C‐reactive protein; FC, fecal calprotectin; Hb, hemoglobin; WBC, white blood cell count.

**Figure:**
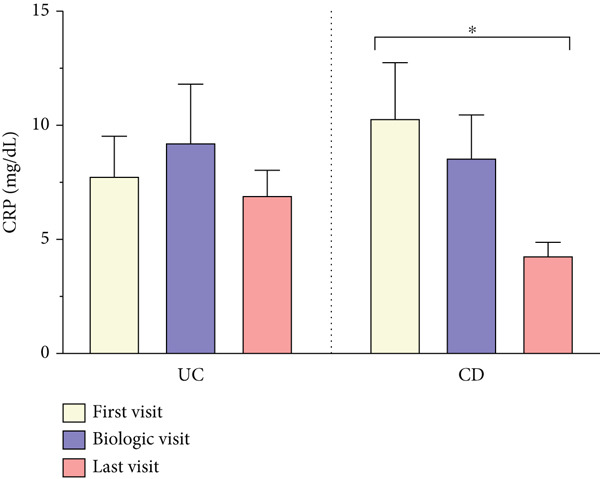
(a)

**Figure:**
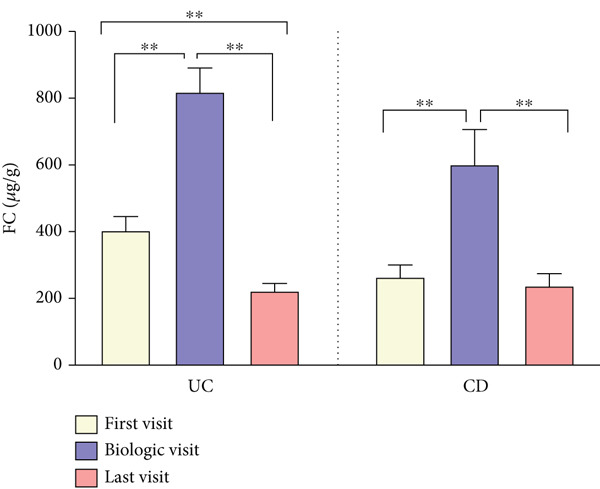
(b)

**Figure:**
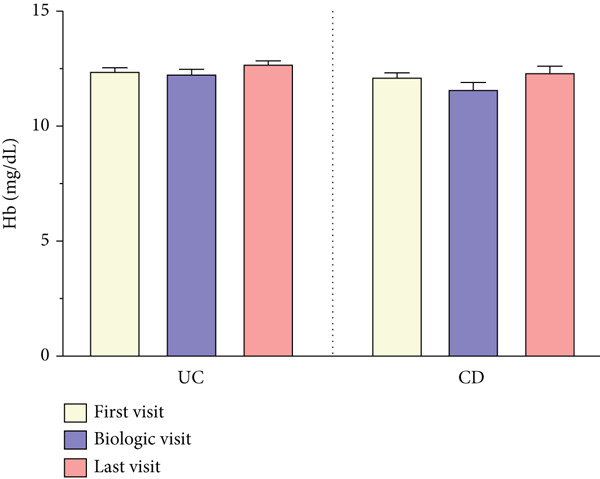
(c)

**Figure:**
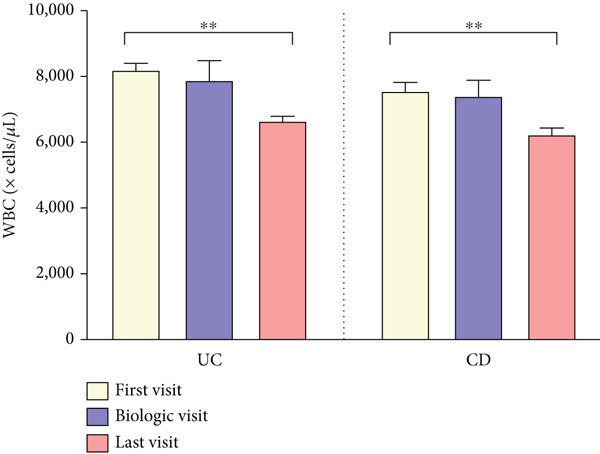
(d)

## 4. Discussion

The present study demonstrated a significantly higher clinical remission rate in patients with UC (96.3%) compared with those with CD (91.2%). A potential reason for our cohort′s higher clinical remission rate may be attributable to the use of novel medications, including biologics. Previous reported remission rates have varied across studies—62% in CD (2007) [20], 71% in UC (2019) [24], and 87.9% in UC (2021) [25]—likely reflecting advances in treatment strategies over time. As disease duration extends, more individualized therapeutic approaches appear to support enhanced clinical improvement.

It is crucial to note that clinical remission may not fully correspond with the treatment goals in IBD. Despite achieving clinical remission, endoscopic evaluations may reveal persistent signs of inflammation. Normal histological status was observed here in only 33.3% of patients with CD and 21.9% of those with UC during endoscopy, despite most being clinically asymptomatic. Evidence suggests that ongoing inflammation, even in symptomatically stable patients, has been associated with poorer long‐term outcomes, including higher risks of hospitalization and surgical interventions [26]. These findings highlight the necessity of incorporating endoscopic assessment, beyond symptom evaluations, to ensure successful management and durable remission in IBD.

Regarding disease location, in this study, ileocolic involvement was the most prevalent in patients with CD (57.4%), while left‐sided colitis was the most frequent extension in patients with UC (41.9%). In a large Iranian IBD population registry study, ileocolic (43.7%) predominated in cases with CD, and pancolitis (47%) was the most frequent type in those with UC [27]. Considerable variation in disease extension has been reported internationally, including ileocolonic involvement in 21%–44% of European patients with CD [28]; left‐sided colitis in 30.6% of Scandinavian patients with UC [29]; ileocolic involvement in 34.4% and left‐sided colitis in 50.5% of Brazilian patients with CD and UC, respectively [25]; and 47.1% with left‐sided colitis and 42.6% with ileocolic disease among Taiwanese patients with UC and CD, respectively [30]. Another European population‐based study reported large variations in disease distribution, about 10%–45% of patients presented with ileocolic disease and 22%–31% with pancolitis [31]. Such variability may suggest the influence of regional or ethnic variations on disease phenotypic and management approaches, highlighting the need for population‐specific diagnostic and therapeutic strategies in IBD care.

The progression to more extensive disease (18%–31% of patients) has been a consistently observed phenomenon across numerous studies [21, 31–34]. Disease progression occurred in 22.1% and 18% of our patients with UC and CD, respectively, while most (95%) significantly regressed to less extensive disease during follow‐up. GI complications in patients with IBD may contribute to disease progression. In this study, GI complications were significantly more frequent in patients with CD (25.7%) than in those with UC (1.6%), with perianal fistula being the most common type, consistent with previous findings [35, 36]. Therefore, prompt recognition and management of GI complications are crucial to prevent disease progression and reduce the risk of serious outcomes. Despite variations in healthcare delivery across different populations, opportunities still exist to enhance disease management. The multifaceted nature of IBD, coupled with the limitations of current treatments, may permit disease progression in some patients, even under optimal care conditions.

At least one relapse occurred in 69.2% and 77% of our patients with UC and CD during the first 5 years of follow‐up, increasing to 86.7% and 91.7%, respectively, after 10 years. These findings were consistent with previous evidence [20, 21, 37] suggesting that chronic intestinal inflammation promotes progressive tissue damage, thereby complicating mucosal healing and increasing relapse risk. Additionally, persistent inflammation can further dysregulate and exaggerate immune responses, triggering subsequent inflammatory episodes. The higher recurrence rate in CD likely reflects its potential to affect any segment of the GI tract and involve all layers of the intestinal wall. Nonetheless, interindividual variability continues to influence disease outcomes.

During follow‐up, 30% of all patients underwent biologic treatment (adalimumab or infliximab), including 54.4% of those with CD and 24% of those with UC (*p* < 0.001). A large Danish population study reported biologic use in 29% of cases with CD and 11% of those with UC [38], while Islam et al. noted higher usage rates—87% and 57%, respectively—during a 10‐year follow‐up [35]. In an Iranian IBD study, 38.4% of patients with CD and 15.2% of patients with UC received biologics [27]. Since UC is typically limited to the mucosa, whereas CD can affect the entire intestinal wall, resulting in fibrotic disease and long‐term complications often requiring surgery [39], timely initiation of biologic treatment in patients with CD, based on disease severity, is crucial for improving long‐term outcomes.

The main limitation of the present study was its retrospective nature, leading to missing clinical data in similar follow‐up durations. Another limitation relates to environmental factors such as diet and lifestyle, which could not be controlled due to the natural course of observation. It is noteworthy that adalimumab has been consistently available in Iran through a local company, which may explain its predominant use in this cohort. Furthermore, infliximab and tofacitinib were the only drugs in their respective classes available at the time.

## 5. Conclusion

This retrospective cohort study showed a high clinical remission rate among patients with IBD, although only one‐third of patients achieved endoscopic remission at maximal follow‐up. The 5‐ and 10‐year relapse rates were high, and severe endoscopic activity was associated with increased relapse frequency during the disease period. Significant phenotypic differences were observed between patients with CD and UC, particularly in initial presentation and treatment strategies. These findings offer valuable insights for optimizing treatment initiation, tailoring therapeutic approaches for subgroups of IBD, and developing personalized follow‐up plans. Further longitudinal and controlled studies are warranted to refine management strategies and improve clinical outcomes for this chronic disease.

## Conflicts of Interest

The authors declare no conflicts of interest.

## Author Contributions

Poria Hoseinialiabadi and Mahboobe Ebrahimi contributed equally to this work.

## Funding

No funding was received for this manuscript.

## Data Availability

The datasets used and/or analyzed during the current study are available from the corresponding author on reasonable request.
